# Identification and molecular evolution of the *GLX* genes in 21 plant species: a focus on the *Gossypium hirsutum*

**DOI:** 10.1186/s12864-023-09524-w

**Published:** 2023-08-22

**Authors:** Menglin Xu, Dongyun Zuo, Qiaolian Wang, Limin Lv, Youping Zhang, Huixin Jiao, Xiang Zhang, Yi Yang, Guoli Song, Hailiang Cheng

**Affiliations:** 1 Zhengzhou Research Base, State Key Laboratory of Cotton Biology, School of Agricultural Sciences, Zhengzhou University, Zhengzhou, 450001 Henan China; 2 State Key Laboratory of Cotton Biology, Cotton Research Institute of Chinese Academy of Agricultural Sciences, Anyang, 455000 Henan China

**Keywords:** Glyoxalase family, Cotton, Phylogeny, Heat treatment

## Abstract

**Background:**

The glyoxalase system includes glyoxalase I (GLXI), glyoxalase II (GLXII) and glyoxalase III (GLXIII), which are responsible for methylglyoxal (MG) detoxification and involved in abiotic stress responses such as drought, salinity and heavy metal.

**Results:**

In this study, a total of 620 *GLX* family genes were identified from 21 different plant species. The results of evolutionary analysis showed that *GLX* genes exist in all species from lower plants to higher plants, inferring that *GLX* genes might be important for plants, and *GLXI* and *GLXII* account for the majority. In addition, motif showed an expanding trend in the process of evolution. The analysis of cis-acting elements in 21 different plant species showed that the promoter region of the *GLX* genes were rich in phytohormones and biotic and abiotic stress-related elements, indicating that *GLX* genes can participate in a variety of life processes. In cotton, *GLXs* could be divided into two groups and most *GLXIs* distributed in group I, *GLXIIs* and *GLXIIIs* mainly belonged to group II, indicating that there are more similarities between *GLXII* and *GLXIII* in cotton evolution. The transcriptome data analysis and quantitative real-time PCR analysis (qRT-PCR) show that some members of *GLX* family would respond to high temperature treatment in *G.hirsutum*. The protein interaction network of GLXs in *G.hirsutum* implied that most members can participate in various life processes through protein interactions.

**Conclusions:**

The results elucidated the evolutionary history of *GLX* family genes in plants and lay the foundation for their functions analysis in cotton.

**Supplementary Information:**

The online version contains supplementary material available at 10.1186/s12864-023-09524-w.

## Background

Abiotic stresses such as drought, salinity and extreme temperature seriously threaten the growth of plants and limit the development of modern agriculture [1–3]. Under abiotic stress, photosynthesis and respiration of plants can produce excessive toxic aldehydes, such as methylglyoxal (MG), glyoxal (GO) and 3-deoxyglucosone (DOG), through enzymatic and non enzymatic pathways [4]. Biomacromolecules will be damaged by higher concentrations of MG, resulting in carbonyl stress. MG can react with arginine, lysine and cysteine to produce irreversible advanced glycation end products (AGEs) [5–7]; MG can also react with membrane lipids to produce irreversible advanced lipid peroxidation end products (ALEs) [8, 9]; In addition, MG can induce the production of ROS, and then generate oxidative stress, which damages proteins, DNA, RNA, lipids and biofilms [9–13]. At low concentration MG can also act as a signal molecule. It forms a signal network through interaction with other signal molecules, such as Ca^2+^, H_2_O_2_, nitric oxide (NO), hydrogen sulfide (H_2_S), to further regulate a variety of physiological processes and stress tolerance such as seed germination, plant growth, development, reproduction [4]. Therefore, it is particularly important to maintain the dynamic balance of MG in cells.

Plants have evolved many effective detoxification mechanisms, including glyoxalase system and non glyoxalase system [14]. The glyoxalase system is the main defense line, accounting for 99% of the total MG clearance [4]. There are three kinds of glyoxalase: glyoxalase I (GLXI), glyoxalase II (GLXII) and glyoxalase III (GLXIII) [15–17]. GLXI removes excess MG with glutathione (GSH) as a cofactor to produce the intermediate S-d lactoylglutathione (SLG), which is then converted into lactic acid by GlXII, and regenerates GSH [4, 18–21]. It can be seen that glyoxalase system can not only remove excess MG to maintain its homeostasis, but also maintain redox homeostasis in cells by regulating GSH regeneration. GLXIII is a glutathione independent glyoxalase, which can directly catalyze the irreversible conversion of MG to lactic acid without the participation of glutathione or other cofactors [22–25].

GLXs are important enzymes for plants to cope with the stress of aldehydes and ketones and abiotic stress [26–29]. So far, there have been some studies on the molecular function of the *GLX* genes, for example, *GLXI* can participate in cell proliferation in soybean [30] and Amaranthus paniculatus [31], be involved in abiotic stress in tomato [32], pumpkin [33], onion [33] and wheat [34], improve stress resistance in tobacco [35], black gram [36], Arabidopsis [37], mustard [38] and rice [39]. *GLXIs* and *GXIIs* can participate in salt stress in rice [40, 41], but not on its molecular evolution. Evolutionary studies of gene families can not only systematically elucidate gene members, sequence structure and molecular functions, but also help to explore biological characteristics of species, such as growth and development, transcriptional regulation and environmental adaptation, laying a solid foundation for biological breeding research [42–45]. In recent years, global water shortage, soil salinization and frequent extreme weather have seriously affected the growth environment of crops [46–50]. Cotton is an important Cash crop in the world, but systematic identification and analysis of *GLX* family in cotton have not been reported yet. In this study, *GLX* genes were identified in different green plants, and an evolutionary analysis was performed. We focused on the identification, evolutionary relationship and function of *GLX* genes in *Gossypium hirsutum.* The results of this study provide a theoretical basis for the evolution and biological functions of the *GLX* genes in plants and the study of *GLX* gene family will help us understand its potential application in cotton.

## Results

### Identification of *GLX* genes in green plants

**Fig. 1:**
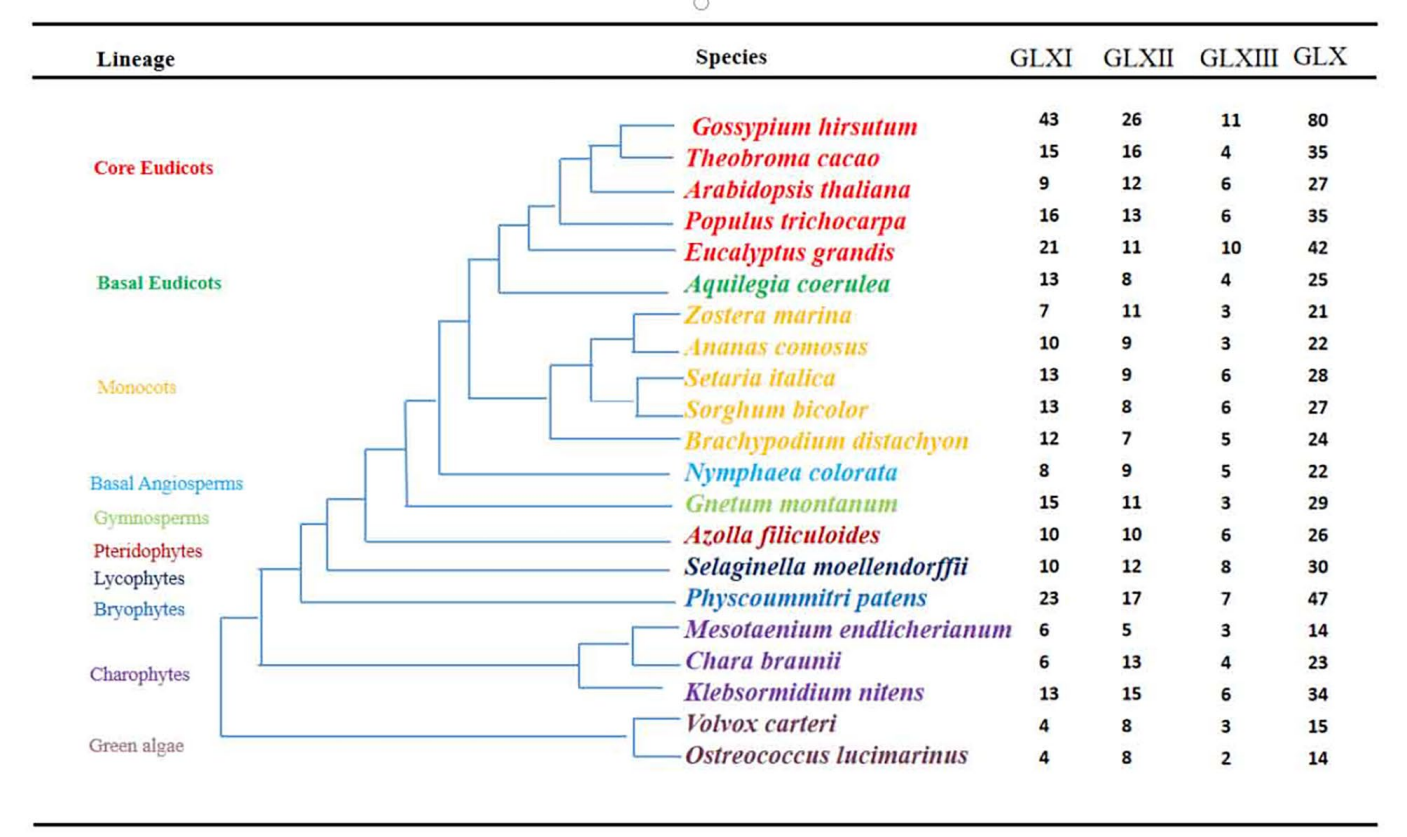
Phylogenetic relationships among 21 plant species. Different background colors in the phylogenetic tree show different lineages. The right side shows the numbers of *GLXI, GLXII*, and *GLXIII* genes in 21 different plant species, and the total number of *GLX* genes in each species

In order to study the evolutionary relationship of *GLX* genes in green plants, 620 *GLX* genes were identified in 21 plant species, which represent the evolutionary relationship of 10 kinds of green plants, including green algae, charophytes, bryophytes, lycophytes, pteridophytes, gymnosperms, basal angiosperms, monocots, basal eudicots, and core eudicots (Fig. 1 and Table S2). The *GLX* gene family has already appeared in green algae, with 14 and 15 *GLX* genes in *O. lucimarinus* and *V. carteri*, respectively. *GLX* genes in other plants were more than green algae (Fig. 1). Notably, from Algae to Bryophytes, there was an explosion of *GLX* number, leading to 47 members in *P. patens*. There were the most *GLX* genes (80) in *G.hirsutum*, which could be due to it is allotetraploid. This indicates that *GLX* family undergo expansion in the process of evolution. Existence of *GLXs* in both lower and higher plants suggested that *GLX* genes could be important for plants. In most species, *GLXI* and *GLXII* are more abundant than *GLXIII*, and gene numbers varied more in different plants. In order to study the homologous domain and preservation degree of GLX protein in evolution, MEME (http://meme-suite.org/tools/meme, accessed on 18 November 2022) was used for motif analysis, and 20 conservative motifs were identified in *GLXI, GLXII*, and *GLXIII* respectively. The results showed that motif generally showed an expansion trend in the evolutionary process. For example, motif 3, motif 9 and motif 15 in *GLXI* were not found in green algae, and gradually appeared from charophytes (Figure S1). In *GLXII*, motif 19 and motif 20 were not found in green algae, and began to appear in charophytes (Figure S2). In *GLXIII*, motif 2, motif 3, motif 8, motif 9, motif 14, motif 16 and motif 20 did not appear in green algae, and began to be found in charophytes with evolution (Figure S3).

### Analysis of putative cisacting elements in *GLX* promoters

**Fig. 2:**
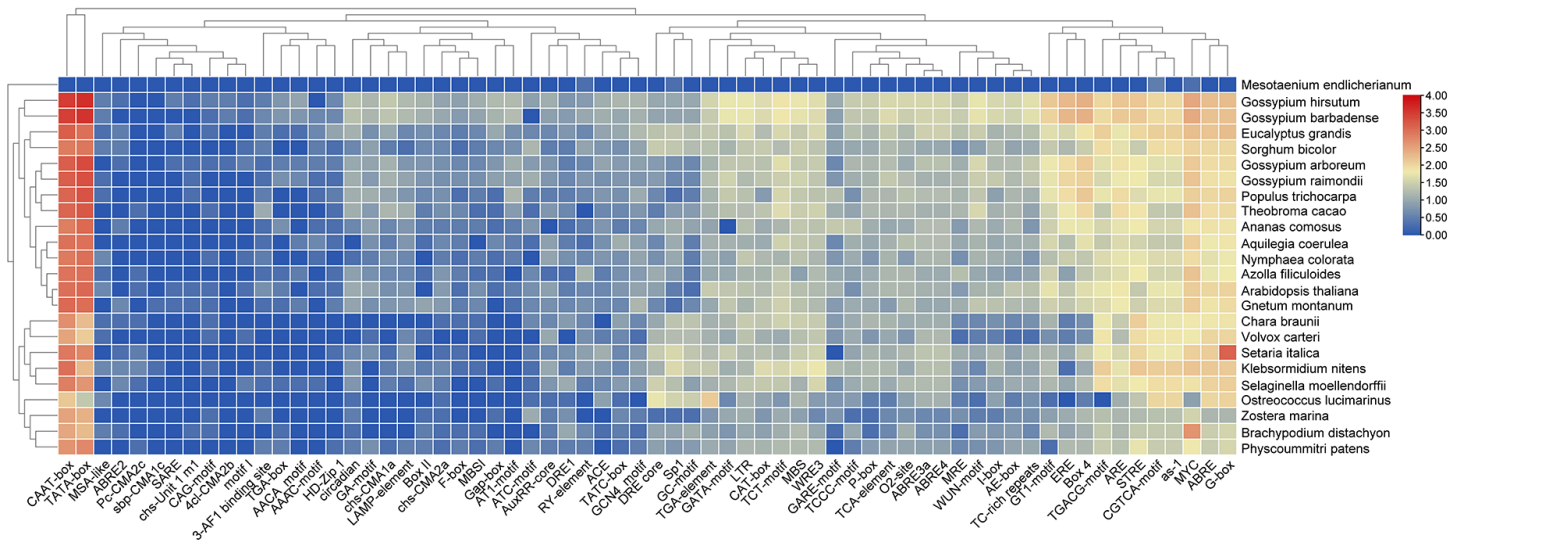
Predicted cis-elements in the promoter regions of *GLX* genes in 21 species. The species names were shown on the right, and the cis-acting elements were displayed at the bottom. Scale bars at the right represented log2 (FPKM + 1). Different colours represent the different numbers of cis-acting elements

The cis-acting element is essentially a DNA sequence, which is a binding site to transcription factors, and regulates the precise initiation and conversion efficiency of gene transcription by binding to transcription factors. The 2-kb promoter sequence of *GLX* genes upstream in 21 plant species was analyzed using PlantCARE database [51]. The results showed that different types of Cis-acting element were found in the *GLX* genes promoter (Fig. 2 and Table S3). As shown in Fig. 2, among them, the core element CAAT box which related to the regulation of nopaline synthase, and the core element TATA box which related to transcription, are the most abundant and exist in most species [52]. Various phytohormone responsive elements have also been found, such as ABRE, an ABA response element [53], ERE, an ethylene-responsive element [54], CGTCA-motif, MYC, chs-CMA1a and TGACG-motif which belong to MeJA-responsive element [55], GARE-box, P-box, TATC-box and Pc-CMA2c which belong to GA-responsive element [56], as-1, SARE, sbp-CMA1c and TCA-element which belong to salicylic acid-responsive element [57], and TGA-element, AuxRR-core and GA-box which belong to IAA-responsive element, this suggests that different phytohormones can regulate the expression of *GLXs*. Various light-related elements such as G-box, Box 4, GT1-motif, GATA-motif, Sp1, I-box, 3-AF1 binding site, TCT-motif, and Gap-box were also observed [58]. The stress-responsive elements were detected in the promoter of some *GLXs*, such as F-box [59], MBS (MYB binding site involved in drought-inducibility), ARE (anaerobic induction element) [60], STRE (stressresponsive element) [61], LTR (low temperature-responsive elements) [62], dehydration-responsive element (DRE1 and DRE-core) [63], TC-rich repeats (defence and stress-responsive element), wound-responsive element (WRE3 and WUN-motif) [64], and GC-motif (enhancer-like element involved in anoxic specific inducibility). Some growth and development elements were also predicted, such as CAT-box (cis-acting regulatory element related to meristem expression), GCN4_motif (cis-regulatory element involved in endosperm expression) [65], circadian (cis-acting regulatory element involved in circadian control) [66] and RY-element (cis-acting regulatory element involved in seed-specific regulation) [67]. The results indicated that *GLXs* are involved in plant growth and development as well as environmental stress responses.

### Phylogenetic analysis of *GLX* genes family in cotton

**Fig. 3:**
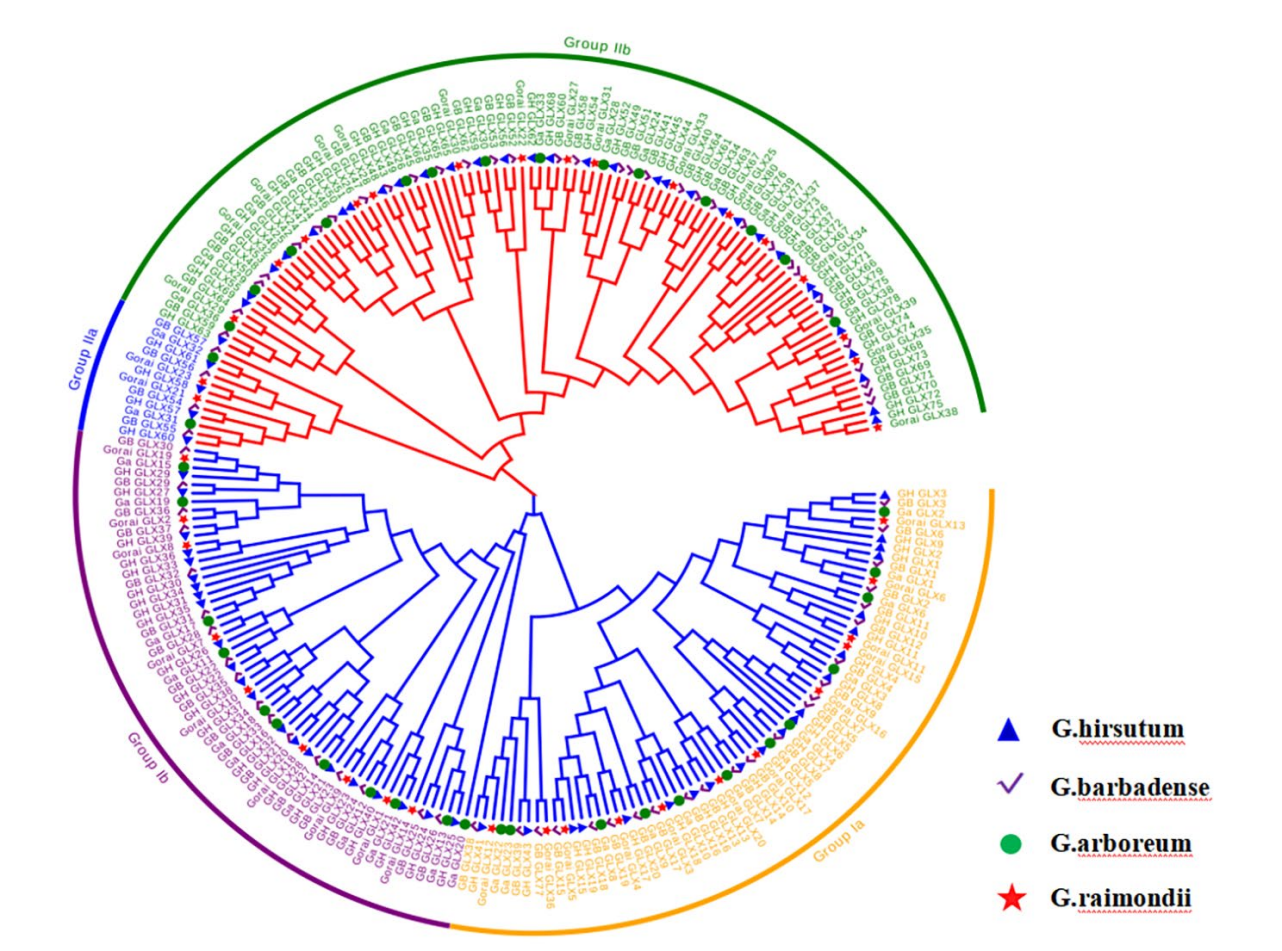
Phylogenetic analysis and subfamily classification of the *GLX* genes in *G. hirsutum* (*GH_GLX*), *G. barbadense* (*GB_GLX*), *G. arboreum* (*Ga_GLX*) and *G. raimondii* (*Gorai_GLX*). The phylogenetic tree was constructed with MEGA 6.0 using the neighbor-joining model with 1000 bootstrap replicates. All 235 *GLXs* in cotton were divided in to four subgroups, which were highlighted by different colors

In order to further study the evolutionary relationship of GLX proteins in cotton. Total 235 *GLX* genes were identified in *G.hirsutum*, *G. arboreum*, *G.raimondii* and *G.barbadense.* Phylogenetic tree was constructed using the amino acid sequences of all the GLX proteins from four cotton species (Fig. 3). The phylogenetic tree showed that all GLX proteins in cotton were divided into two groups. The first group consists of 127 members and is divided into two subgroups. The second group was divided into two subgroups and contained 108 members. All GLXI are distributed in group I, GLXII and GLXIII are mostly distributed in Group II, indicating that the members of GLXII and GLXIII are more similar in evolution.

### Collinearity analysis of the G*LX* gene family in cotton

**Fig. 4:**
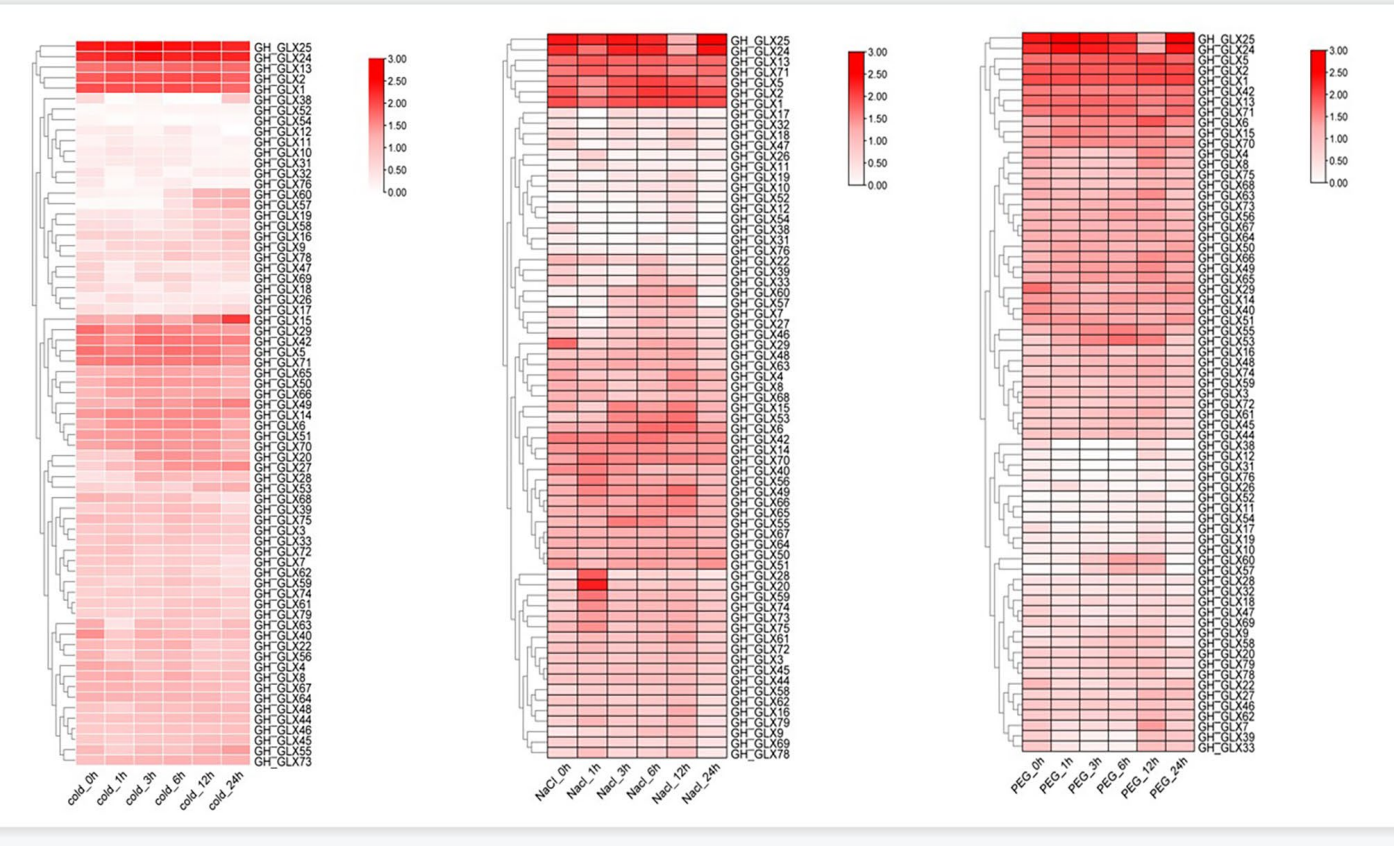
The sub-genome distribution and synteny analysis of cotton *GLX* genes. The blue lines represents duplicated *GLX* pairs, the gray lines represents collinear blocks

The *G. hirsutum* and *G.barbadense* are evolved due to hybridization between an A-genome species (ancestor of *G.herbaceum* or *G.arboreum*) and a D-genome species (ancestor of *G.raimondii*) [68]. To understand the evolutionary relationships of *GLX* genes in cotton, a relative syntenic map of *GLX* genes from the four cotton species was fabricated (Fig. 4). According to MCScan analysis, 103 duplicate gene pairs were found between diploid *G. arboreum* and tetraploid *G.hirsutum*, and also 103 between diploid *G.arboreum* and tetraploid *G.barbadense*. Meanwhile, 113 and 112 duplication gene pairs were found between diploid *G.raimondii* and tetraploid *G.hirsutum*, diploid *G.raimondii* and tetraploid *G.barbadense*, respectively. The *GLX* genes of *G.Aimondii* has a good collinear relationship with the *GLX* genes of *G.hirsutum* and *G.barbadense* on chr03, chr09, chr10 and chr11. The *GLX* genes of *G.arboreum* has a good collinear relationship with the *GLX* genes of *G.hirsutum* and *G.barbadense* on chr10. The result shows that during the evolution of *GLXS*, chromosome may have undergone small deletion, duplication and reshuffling [69].

### Expression analysis of *GLX* genes under stress in *G.hirsutum*

**Fig. 5:**
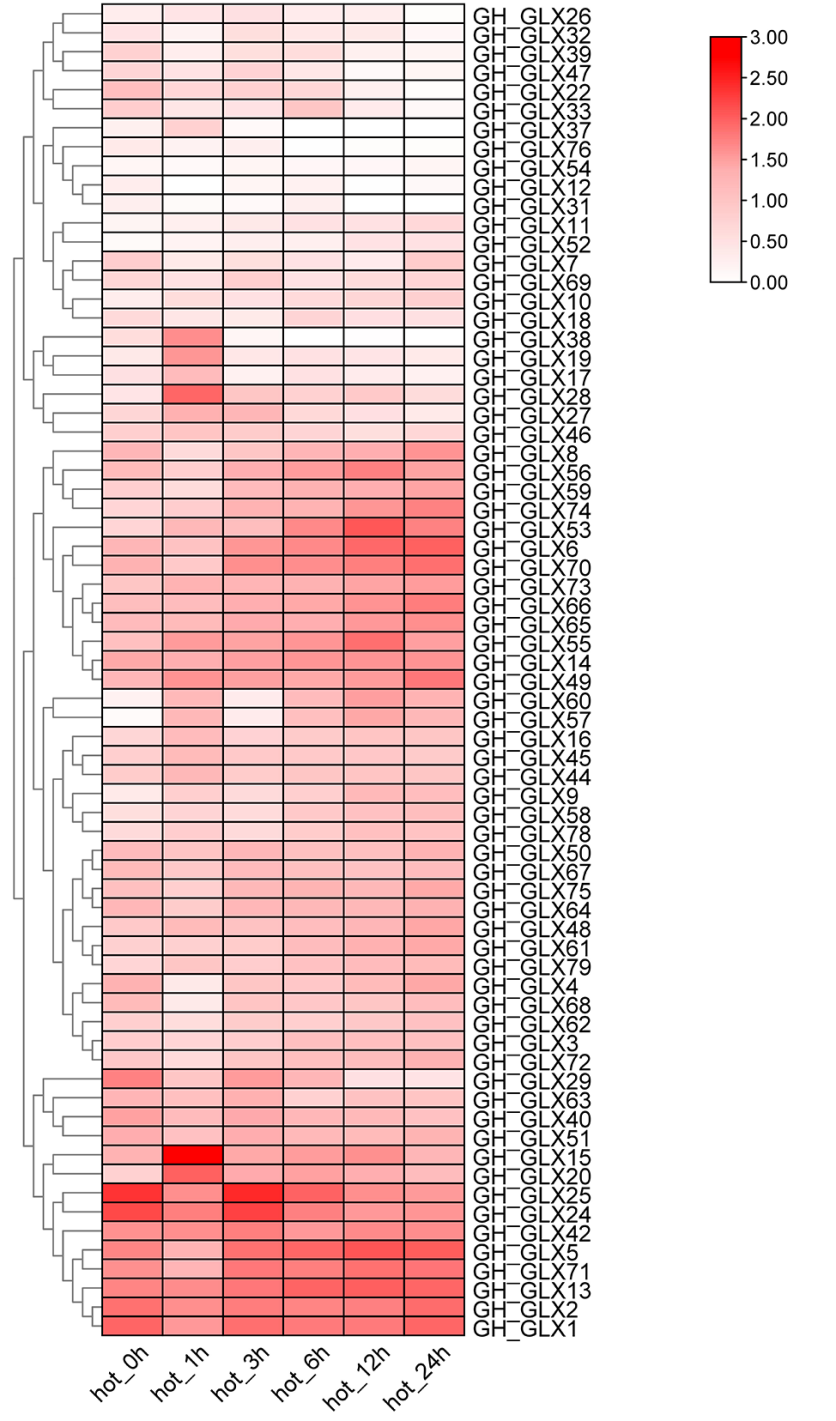
Expression of *GLX g*enes in *G.hirsutum* under heat treatment. The phylogenetic relationships were displayed on the left, the gene names were shown on the right, and the time after heat treatment is displayed at the bottom. Scale bars at the right represented log2 (FPKM+ 1). Different colours represent the different expression levels of *GH_GLXs*

In previous studies, it was reported that members of the *GLX* family were associated with stress response in rice. In order to explore whether the *GLXs* of *G. hirsutum* were also related to stress responses, gene expression profiles after stresses treatment were obtained from public RNA-seq data. It can be seen that the expression levels of *GLX15*, *GLX19*, *GLX20*, *GLX24*, *GLX25*, *GLX28* and *GLX38* changed under 37℃ treatment (Fig. 5, Figure S4 and Table S4), suggesting that these seven genes may respond to high temperature.

**Fig. 6:**
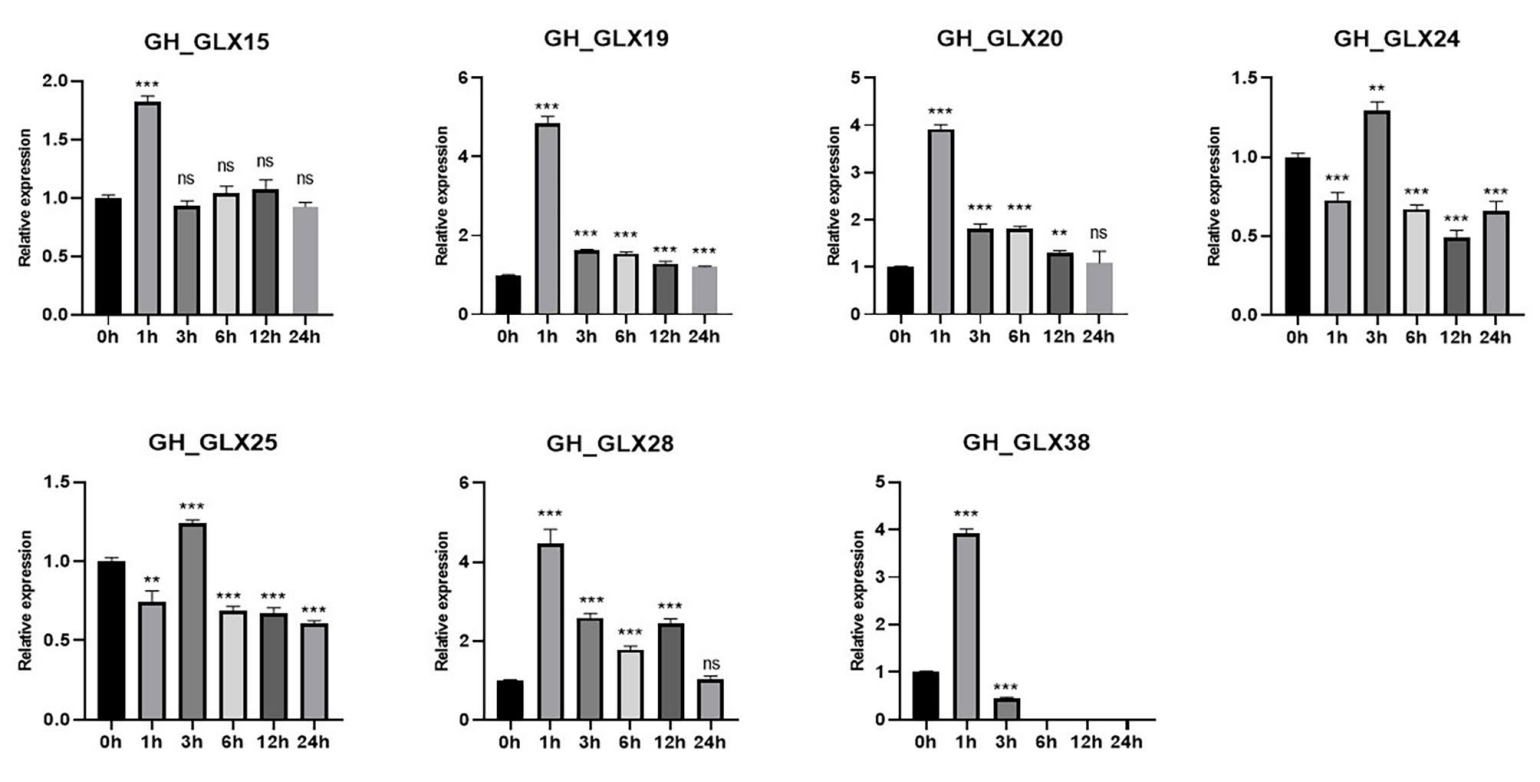
The expression patterns of 7 *GH_GLXs* at different time after heat treatment by qRT-PCR. The values are standardized. *GhUBQ7* (DQ116441) was used as the internal control. Each experimnet was performed in three biological replicates, and the error bars represent mean ± SD; n = 3. (**p < 0.01, ***p < 0.001, Student’s *t*-test)

*GLX20*, *GLX28* and *GLX38* have the highest expression level in anthers, *GLX19* is mainly expressed in roots, *GLX15* is mainly expressed in leaves and roots, and *GLX24* and *GLX25* are mainly highly expressed in leaves(Figure S5 and Table S5). To validate the expression change of 7 *GLX* genes under heat treatment, qRT-PCR was performed to verify their expression in leaves at 0 h, 1 h, 3 h, 6 h, 12 h, and 24 h after heat treatment. The qRT-PCR results were almost consistent with transcriptome data. The trend of *GLX15*, *GLX19*, *GLX20*, *GLX28* and *GLX38* was roughly the same, the expression levels of all genes were the highest at 1 h after heat treatment, and began to decrease significantly at 3 h, and then almost leveled off, *GLX38* was almost not expressed after 3 h treatment. The expression trend of *GLX24* and *GLX25* was almost the same, the expression decreased after 1 h heat treatment, reached the highest level at 3 h, and then tended to be stable. In short, the expression of these seven genes roughly decreased with the passage of heat treatment time, and responded to heat treatment (Fig. 6).

### Prediction of GLX protein interactions in *G.hirsutum*

**Fig. 7:**
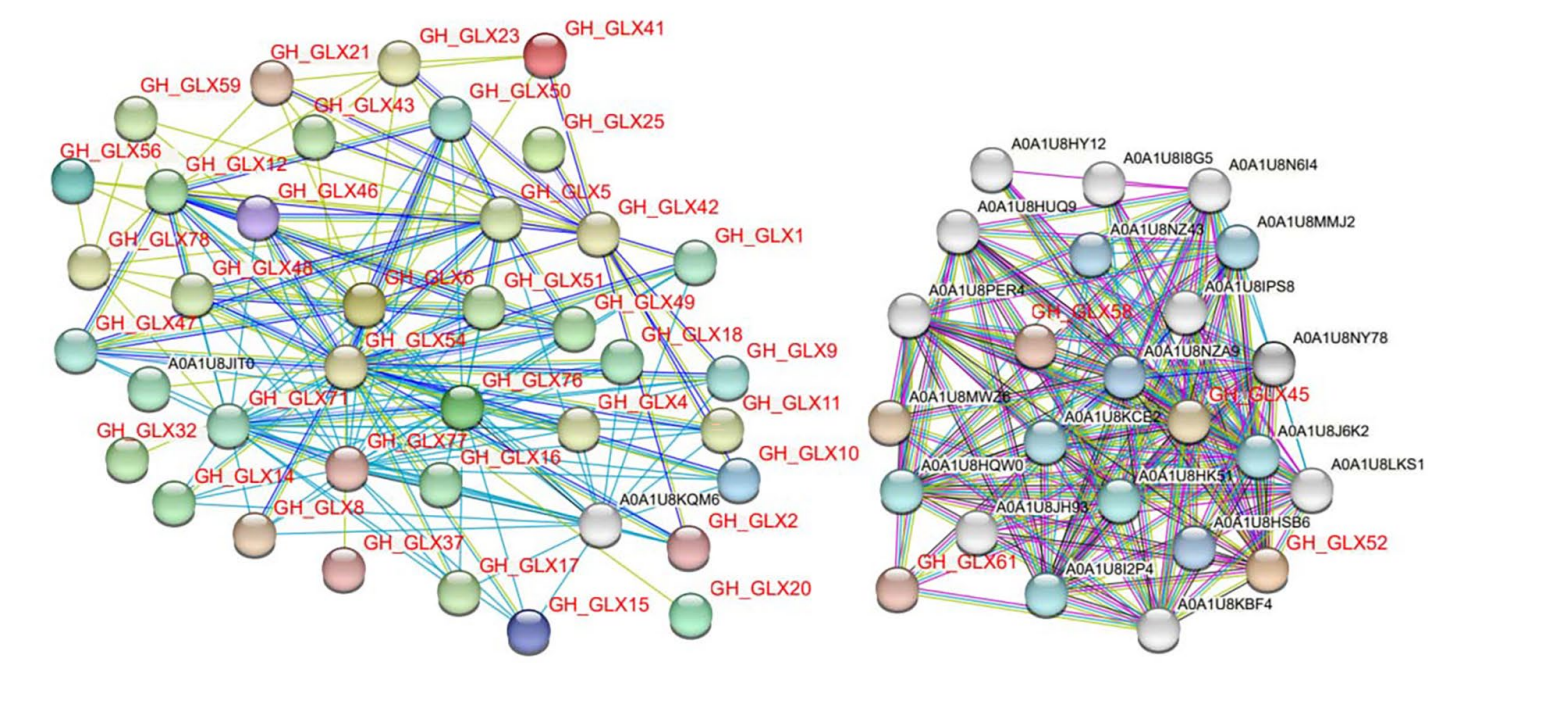
Functional network assembly of the GLX proteins in *G.hirsutum*. Red letters represent the GLX proteins. Light blue and purple lines stand for represent known interactions determined by the database and experiments, respectively. Green, red, and blue lines stand for predicted interactions from gene proximity, fusion, and symbiosis, respectively. Light green, black, and gray lines represent other interactions from text mining, co-expression, and protein homology, respectively. Empty nodes: proteins of unknown three-dimensional structure

To further detect the potential roles of GLX family members, we investigated the interaction of the GLX proteins in *G.hirsutum* using the STRING database [70]. The results showed that most members of GLX family can interact with each other, and a few could interact with other proteins (Fig. 7). For instance, GLX61 and GLX58 can interact with A0A1U8KBF4, A0A1U8HUQ9, A0A1U8PER4 and A0A1U8NZA9 which related to cleavage. GLX52 and GLX45 can interact with A0A1U8N6I4, A0A1U8KCE2, A0A1U8HY12 and A0A1U8J6K2, which are related to segmentation, flowering time, nucleotide polymerization and phosphorylation, respectively. This implies that GLX proteins may function as homologous protein complex and participate in various biological processes through protein interactions.

## Discussion

Glyoxalase was discovered in animal in 1913 and not reported in plants until the end of the 20th century [28]. In plants, the *GLX* gene family has an important impact on growth, development and coping with abiotic stresses [71]. In this study, a total of 620 *GLX* genes were identified in 21 green plants, and *GLX* genes were distributed in all the 21 species, but the number of *GLXI* and *GLXII* in each species was far greater than that of *GLXIII* (Fig. 1 and Table S2), this indicates that *GLX* genes are widely distributed in plants, but the number of *GLXIII* is relatively small.

The promoter is DNA sequence located in the upstream of the structural gene and recruit RNA polymerase for transcription initiation [72]. The specificity of gene expression depends on cis-regulatory elements [73]. Therefore, 2000 bp promoter sequences of all the *GLX* genes were extracted, and the cis-acting elements were predicted using the PlantCRAE. Various types of elements were found in the promoter sites of *GLX* genes in green plants. The distribution of cis-acting elements among different species was different, as shown in a large number of cis-acting elements were predicted to be related to transcription, various hormones, and stresses response, cell cycle, and development. The results indicated that *GLXs* are might involved in plant growth and development as well as environmental stress responses (Fig. 2).

Cotton is one of the most important cash crops in the world. At the same time, cotton is also a crop with strong drought resistance and salt tolerance, which is suitable for planting in moderate drought conditions or mild saline-alkali land. Based on this characteristic of cotton, we mainly studied *GLX* gene in cotton. A total of 235 *GLXs* family members were identified in four cultivated cotton species, and the phylogenetic tree divided all *GLXs* into two groups, *GLXI* distributed in the first subgroup, *GLXII* and *GLXIII* mostly distributed in the second subgroup (Fig. 3). This suggests that *GXLII* and *GLXIII a*re relatively close in evolution. According to previous studies, although *GLXII* and *GLXIII* are close in evolution, there are still great differences in function. In terms of function, *GLXII* and *GLXI* have similar modes of action, both catalyzing cascade reactions [18–21]. Unlike *GLXI* and *GLXII*, *GLXIII* can directly convert MG irreversibly into non-toxic lactic acid without glutathione glyoxylase [22–25]. The relative syntenic map of *GLX* genes from the four cotton species indicate that chromosome may have undergone small deletion, duplication and reshuffling during the evolution of *GLXs* (Fig. 4).

The potential role of the *GLX* family members was further explored by predicting *G.hirsutum* transcriptome data, quantitative real-time PCR analysis (qRT-PCR) and protein interaction networks. The results showed that the expression levels of *GLX15*, *GLX19*, *GLX20*, *GLX24*, *GLX25*, *GLX28*, and *GLX38* changed under high temperature treatment, indicating that they were responsive to high temperature treatment (Figs. 5 and 6). It was also found that the expression levels of these seven genes were different in different tissues. *GLX20*, *GLX28*, and *GLX38* were mainly expressed in anthers, *GLX19* was highly expressed in roots, *GLX15* was expressed in leaves and roots, and *GLX24* and *GLX25* have high expression level mainly in leaves. (Figure S4 and Table S5). This indicates that although *GLX* exists widely in nature, its expression levels in different tissues and organs are different, which is consistent with the results of previous studies. Meanwhile, the protein interaction prediction indicated that most *GLX* genes interact with each other. *GLX61*, *GLX58*, *GLX52* and *GLX45* may be involved in cleavage, flowering time, nucleotide polymerization and phosphorylation by different protein interactions (Fig. 7).

## Conclusion

In this study, 620 *GLX* genes were identified from 21 plants. It was found that *GLX* genes are distributed in lower and higher plants and widely exist in nature, but their expression levels are different in different tissues and organs. We focused our analysis on *GLX* genes in cotton and found that some of them might be involved in abiotic stress and some other life processes through interacting proteins.

## Materials and methods

### Genome-wide identification of *GLX* family genes in plants

In this study, the genome-wide data of 21 plant species were analyzed. The genomic, CDS and protein sequences of *G. hirsutum* (ZJU), *G. arboreum* (CRI), *G. barbadense* (ZJU) and *G.raimondii* (JGI) were downloaded from CottonGen (https://www.cottongen.org, accessed on 18 November 2022) [74–76]. The genomic, CDS and protein sequences of Arabidopsis was downloaded from TAIR (https://www.arabidopsis.org/, accesses on 18 November 2022) [77]. The genomic, CDS and protein sequences of *Klebsormidium nitens* and *Theobroma cacao* were downloaded from NCBI. The sources of genomic data for others species are listed in Table S1. *GLXI* genes domain information (PF00903, PF12681), *GLXII* genes domain information (PF00753), and *GLXIII* genes domain information (PF01965) were downloaded from Pfam database(https://pfam.xfam.org, accesses on 18 November 2022) [78], Then, we used HMMER3.0 software (http://www.hmmer.org/, accessed on 18 November 2022) with an e-value of 1 × 10^− 5^ as the threshold to acquire GLX protein sequence. Use online tool SMART (http://smart.embl-heidelberg.de/, accessed on 18 November 2022) to further analyze the domain, remove the sequence without *GLX* domain, and finally obtain all genes of *GLX* family [79].

### Analysis of Cis-acting elements in the promoter regions of *GLX* genes

A 2.0 kb of promoter sequence upstream from the transcription start site in each *GLX* genes were extracted from the genome database and analyzed using PlantCare (http://bioinformatics.psb.ugent.be/webtools/plantcare/html, accesses on 18 November 2022) online software to predict the putative cis-acting regulatory elements [51]. After classifying and counting the results [80], TBtools software was used for visualization [81].

### Construction of phylogenetic tree of *GLX* family in cotton

Multi-sequence alignment of all GLX protein sequences was carried out using Clustal X [82], and phylogenetic trees were constructed using the MEGA software proximity method (version 6.0) (Neighbor-Joining, NJ) [83] with the calibration parameter Bootstrap being set to 1000. The online software Evolview (https://www.omicsclass.com/article/671, accessed on 10 July 2021) was used to modify the evolutionary tree [84].

### Collinearity analysis of the *GLX* genes family in cotton

The gene duplication analysis of four cotton species were conducted using MCScanX software [85]. The synonymous substitution (Ks) and nonsynonymous substitution (Ka) of each duplicated gene pairs were calculated in TBtools with default parameters [81]. Gene duplication and synteny relationship were visualized by TBtools [81].

### Expression of *GLX* genes under heat treatment in *G.hirsutum*, Sample collection, RNA extraction, cDNA synthesis, and qRT-PCR analysis

Transcriptome data were downloaded from the Cotton Omics Datebase (http://cotton.zju.edu.cn/10.rnasearch.html, accessed on 18 November 2022). The heat map generated by TBtools software is used to display the relative expression level [81]. Upland cotton cultivar Z12 was planted under controlled conditions. Heat treatment was applied to plants at three leaf stage. Leaves were collected from treated plants after 0 h, 1 h, 3 h, 6 h, 12 and 24 h of treatment. Three biological repeats were collected in each stage. All samples were immediately frozen in liquid nitrogen and stored at -80 °C. Plant total RNA was extracted by the RNAprep Pure Plant Plus Kit (Polysaccharides&Polyphenolicsrich) (DP441) (Tiangen, Beijing, China). The reverse transcription kit was the Prime Script™ II 1st Strand cDNA Synthesis Kit (Genstar, Beijing, China). It was used to reverse the extracted RNA to obtain the first-strand cDNA for transcriptomic and qRT-PCR analysis. Specific primers were designed according to the CDS sequence of the *GH_GLX* genes on the primer-blast of NCBI website (Table S6). *GhUBQ7* (DQ116441) was used as the internal control. qRT-PCR was performed with LightCycler 480 Real-Time PCR system (Roche, Switzerland). Obtaining gene expression data using three biological replicates. Moreover, the relative quantitative analysis of gene expression was carried out by the 2^−∆∆ Ct^ method with three independent replicates [86].

### Prediction of GLX protein interactions in *G.hirsutum*

The prediction of GLX protein interactions was established using the STRING database (https://cn.string-db.org/, accessed on 18 November 2022) [70], Parameter uses the default values.

### Electronic supplementary material

Below is the link to the electronic supplementary material.

Additional file: Figure S1. Motif analysis of GLXI gene in 21 plant species

Additional file: Figure S2. Motif analysis of GLXII gene in 21 plant species

Additional file: Figure S3. Motif analysis of GLXIII gene in 21 plant species

Additional file: Figure S4. Expression of GLX genes under abiotic stress in *G.hirsutum*

Additional file: Figure S5. Tissue-specific expression of seven differentially expressed genes

Additional file: Table S1. The genome data sources for the species used in this study

Additional file: Table S2. GLX gene IDs in 21 plant species

Additional file: Table S3. List of identified cis-elements in the putative promoter region of *GLX* genes

Additional file: Table S4. Expression of GLX genes under abiotic stress in *G.hirsutum*

Additional file: Table S5. Tissue-specific expression of seven differentially expressed genes

Additional file: Table S6. List of primers used in qRT-PCR experiments

## Data Availability

The following information was supplied regarding data availability: Data is available at NCBI SRA, accession numbers: PRJNA490626(https://www.ncbi.nlm.nih.gov/sra?linkname=bioproject_sra_all&from_uid=490626) and PRJNA248163 (https://www.ncbi.nlm.nih.gov/sra?linkname=bioproject_sra_all&from_uid=248163). Extra data has been appended as supplementary Tables.
