# Supplementary figures and images for: Identification and molecular evolution of the GLX genes in 21 plant species: a focus on the Gossypium hirsutum
Source: BMC Genomics. 2023 Aug 22;24:474. doi: 10.1186/s12864-023-09524-w (PMC10464159; doi:10.1186/s12864-023-09524-w)

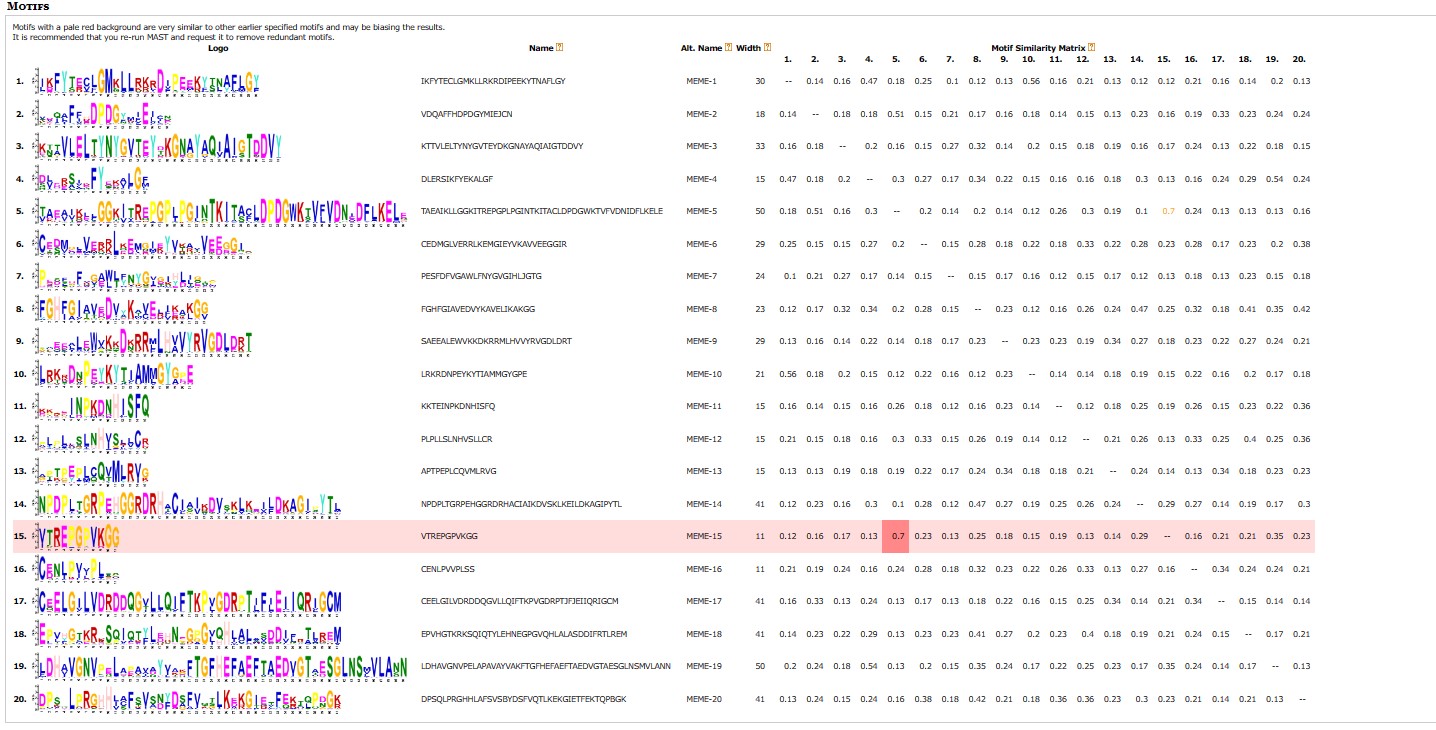

Supplement: Supplementary file 1 — Additional file: Figure S1. Motif analysis of GLXI gene in 21 plant species [file 12864_2023_9524_MOESM1_ESM.jpg]

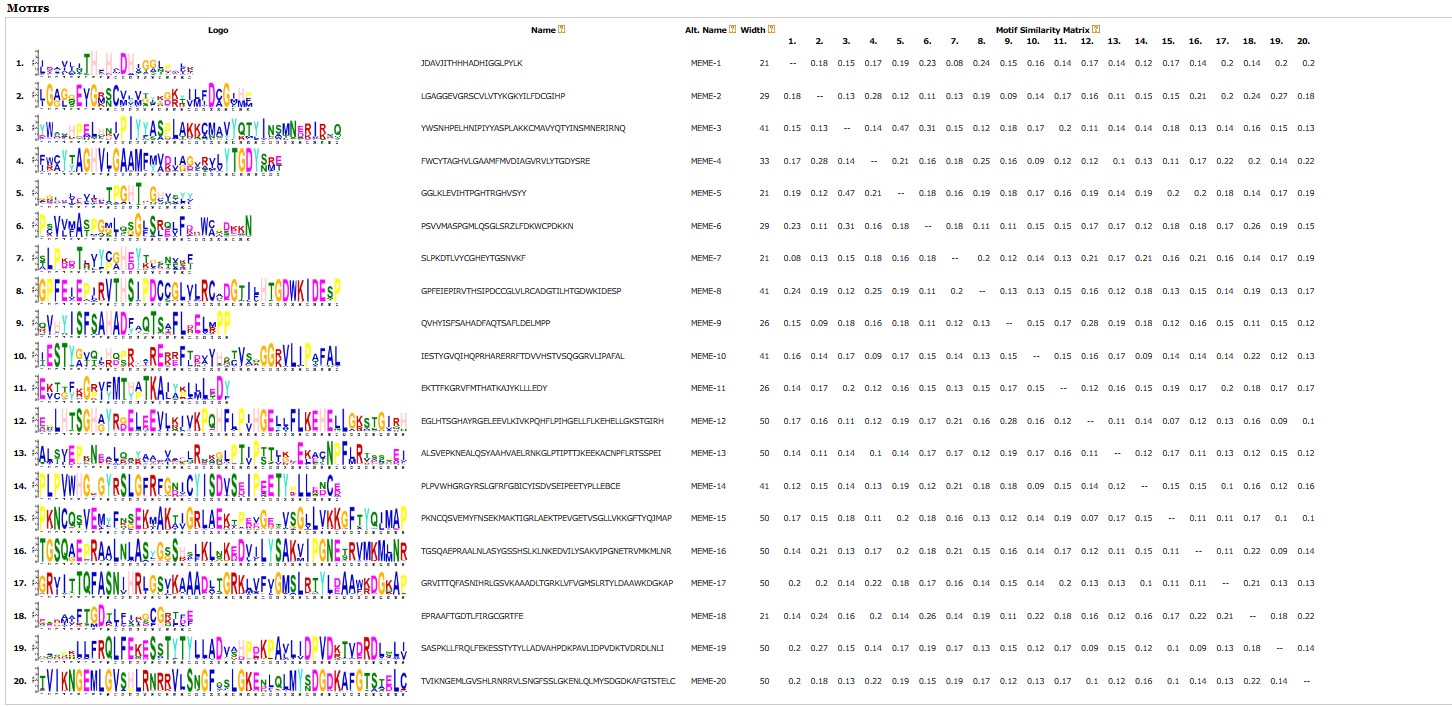

Supplement: Supplementary file 2 — Additional file: Figure S2. Motif analysis of GLXII gene in 21 plant species [file 12864_2023_9524_MOESM2_ESM.jpg]

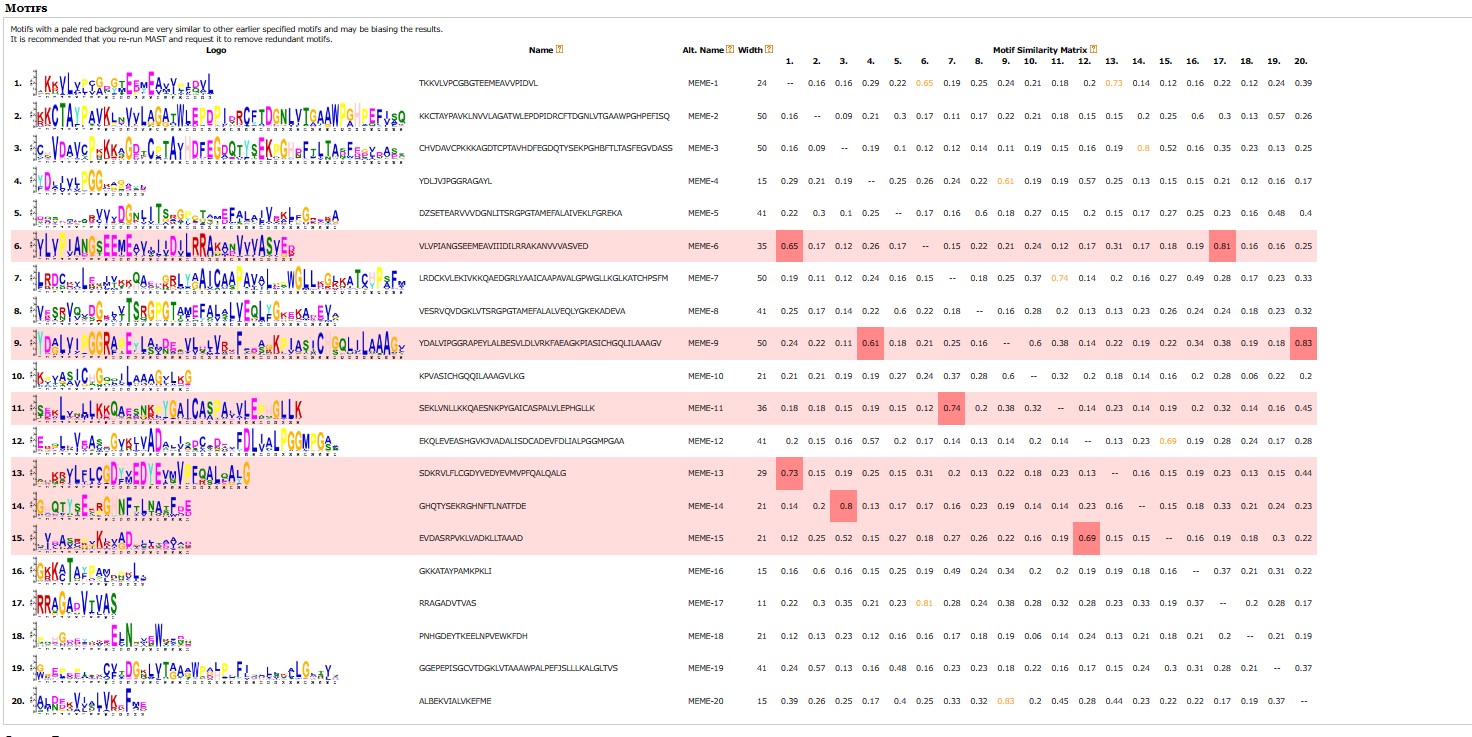

Supplement: Supplementary file 3 — Additional file: Figure S3. Motif analysis of GLXIII gene in 21 plant species [file 12864_2023_9524_MOESM3_ESM.jpg]

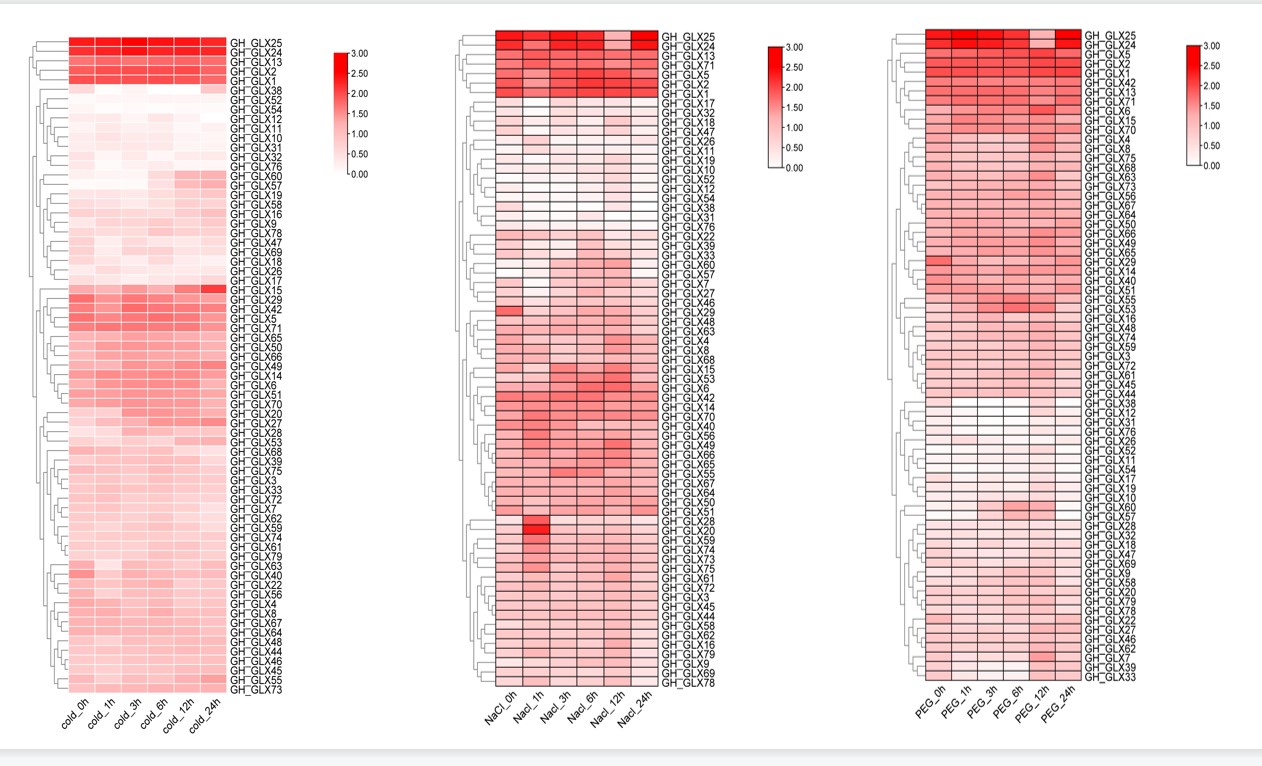

Supplement: Supplementary file 4 — Additional file: Figure S4. Expression of GLX genes under abiotic stress in G.hirsutum [file 12864_2023_9524_MOESM4_ESM.jpg]

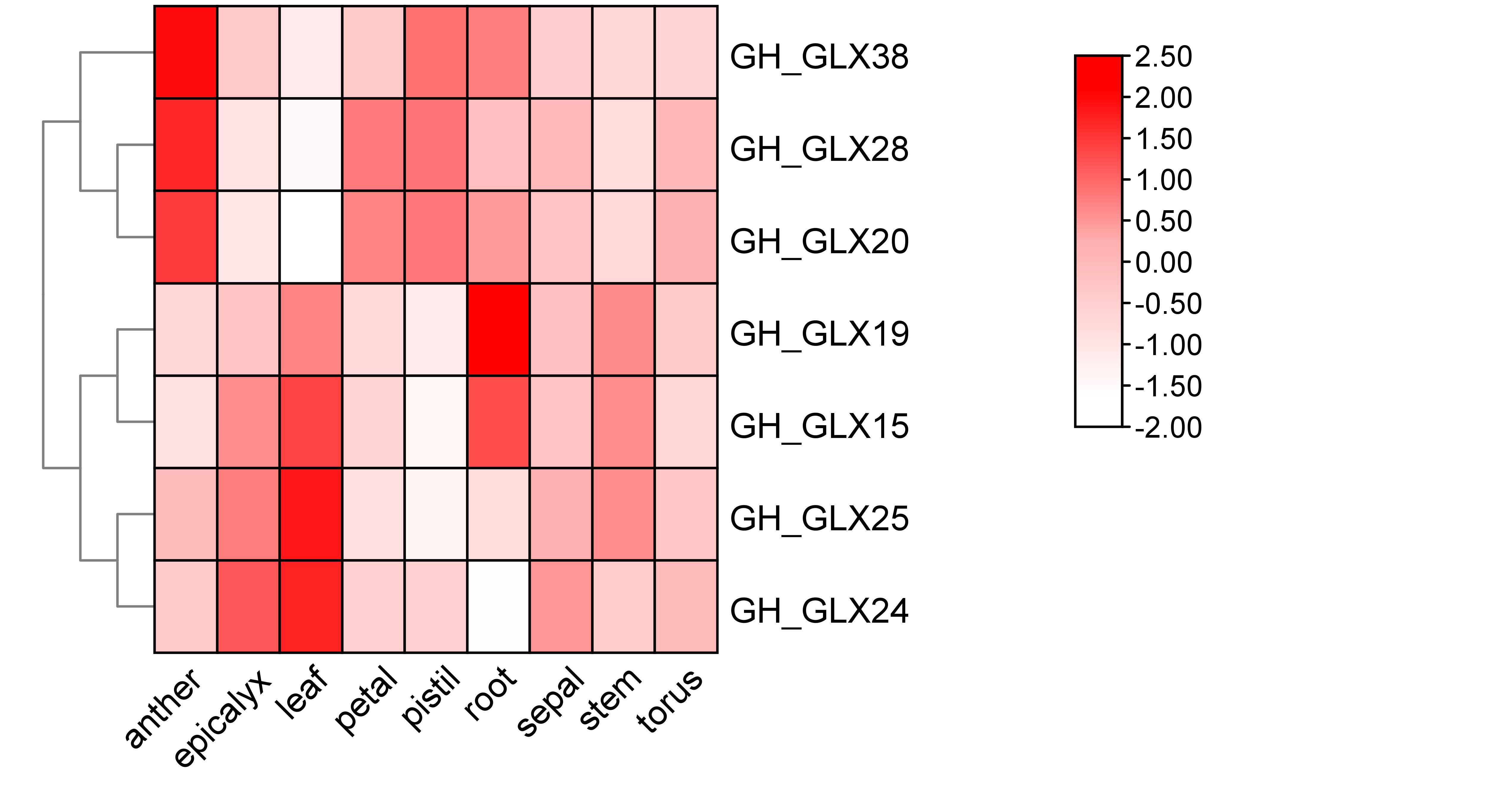

Supplement: Supplementary file 5 — Additional file: Figure S5. Tissue-specific expression of seven differentially expressed genes [file 12864_2023_9524_MOESM5_ESM.jpg]
